# Functional coupling networks inferred from prefrontal cortex activity show experience-related effective plasticity

**DOI:** 10.1162/NETN_a_00014

**Published:** 2017-10-01

**Authors:** Gaia Tavoni, Ulisse Ferrari, Francesco P. Battaglia, Simona Cocco, Rémi Monasson

**Affiliations:** Laboratoire de Physique Statistique, Ecole Normale Supérieure, PSL Research and CNRS - UMR 8550, Paris Sorbonne UPMC, Paris, France; Laboratoire de Physique Théorique, Ecole Normale Supérieure, PSL Research and CNRS- UMR 8549, Paris Sorbonne UPMC, Paris, France; Donders Institute for Brain, Cognition and Behaviour, Radboud Universiteit, Nijmegen, The Netherlands

**Keywords:** Cell assemblies, Ising model, Statistical inference, Effective plasticity, Memory consolidation

## Abstract

Functional coupling networks are widely used to characterize collective patterns of activity in neural populations. Here, we ask whether functional couplings reflect the subtle changes, such as in physiological interactions, believed to take place during learning. We infer functional network models reproducing the spiking activity of simultaneously recorded neurons in prefrontal cortex (PFC) of rats, during the performance of a cross-modal rule shift task (task epoch), and during preceding and following sleep epochs. A large-scale study of the 96 recorded sessions allows us to detect, in about 20% of sessions, effective plasticity between the sleep epochs. These coupling modifications are correlated with the coupling values in the task epoch, and are supported by a small subset of the recorded neurons, which we identify by means of an automatized procedure. These potentiated groups increase their coativation frequency in the spiking data between the two sleep epochs, and, hence, participate to putative experience-related cell assemblies. Study of the reactivation dynamics of the potentiated groups suggests a possible connection with behavioral learning. Reactivation is largely driven by hippocampal ripple events when the rule is not yet learned, and may be much more autonomous, and presumably sustained by the potentiated PFC network, when learning is consolidated.

## Author Summary

Cell assemblies coding for memories are widely believed to emerge through synaptic modification resulting from learning, yet their identification from activity is very arduous. We propose a functional-connectivity-based approach to identify experience-related cell assemblies from multielectrode recordings in vivo, and apply it to the prefrontal cortex activity of rats recorded during a task epoch and the preceding and following sleep epochs. We infer functional couplings between the recorded cells in each epoch. Comparisons of the functional coupling networks across the epochs allow us to identify effective potentiation between the two sleep epochs. The neurons supporting these potentiated interactions strongly coactivate during the task and subsequent sleep epochs, but not in the preceding sleep, and, hence, presumably belong to an experience-related cell assembly. Study of the reactivation of this assembly in response to hippocampal ripple inputs suggests possible relations between the stage of behavorial learning and memory consolidation mechanisms.

In recent years, many techniques have been developed to monitor brain activity in a detailed way and provide a multichannel, multidimensional picture. Different channels may represent coarse-grained activity of neurons in ∼ 1 mm^3^ volumes like in functional magnetic resonance imaging (fMRI) (Friston, 2011), or give access to the activity of single neurons, such as with multielectrode electrophysiological recording (McNaughton, O’Keefe, & Barnes, 1983; Meister, Pine, & Baylor, 1994; Nicolelis, 2007) or calcium imaging (Chhetri et al., 2015; Nguyen et al., 2016; Wolf et al., 2015). The development of these techniques was motivated by the need to characterize the brain state at the network level, and to understand how connections between neurons determine the dynamics and the information processing of neural ensembles. While much progress has been made in characterizing the *connectome*, that is, the exact pattern of connections between neurons in a brain circuit (Seung, 2011), the link between this anatomical architecture and neural network function remains elusive, at least for large-scale circuits. A useful notion in this context is that of *functional couplings*. Functional couplings are generally derived by a reverse inference procedure (Stevenson, Rebesco, Miller, & Körding, 2008): Connections between neurons are assumed to determine neural activity, by way of a statistical or dynamical model, and the coupling values are calculated as those most likely to produce the observed activity data. Compared with earlier estimates of functional interactions based on activity correlations (Aertsen, Gerstein, Habib, & Palm, 1989; Fujisawa, Amarasingham, Harrison, & Buzsáki, 2008; Gerstein & Perkel, 1969; Schwindel, Ali, McNaughton, & Tatsuno, 2014), reverse inference techniques have the advantage of discounting correlations due to interaction paths going through third-party neurons between the recorded cells, therefore providing a much sharper picture of the underlying interactions. While functional couplings are not likely to match *one-on-one* structural couplings, the fingerprint of neural interactions they provide could be used to track changes in the underlying connectivity. A central paradigm in neuroscience is that couplings are plastic: Learning and memory consolidation happen through changes in neural synaptic couplings following repeated coincident pre- and post-synaptic activations, as was postulated by D. Hebb and proven by in vitro experiments showing long-term potentiation (LTP) and long-term depression (LTD) after repeated coincident and non–coincident stimulations (Bliss & Collingridge, 1993; Castillo, 2012).

Hebb further postulated that cell assemblies (Buzsáki, 2010; Harris, Csicsvari, Hirase, Dragoi, & Buzsáki, 2003; Hebb, 1949), closely connected, synchronously activating groups of cells, are the main constituents of memory and information representations. The activation and reactivation (“replay”) of cell assemblies is thought to be critical for consolidation and re-elaboration of memories, working memory, and decision-making (Carr, Jadhav, & Frank, 2011; O’Neill, Pleydell-Bouverie, Dupret, & Csicsvari, 2010; Wilson & McNaughton, 1994). The precise characterization of cell assemblies from experimental data remains, however, very difficult. Current available methods for cell assembly detection and replay estimation often rely on the identification and the matching of templates (Johnson & Redish, 2007; Pfeiffer & Foster, 2013; Singer, Carr, Karlsson, & Frank, 2013). In the hippocampus, for instance, such templates are provided by the temporal sequence of firing events of place cells during the awake phase. The pairwise cross-correlation matrix can also be used to search for clusters of neurons with related firing patterns (Billeh, Schaub, Anastassiou, Barahona, & Koch, 2014; Lopes-dos-Santos, Ribeiro, & Tort, 2013), or to approximate templates from principal component analysis (PCA) (Peyrache, Benchenane, Khamassi, Wiener, & Battaglia, 2010; Peyrache, Khamassi, Benchenane, Wiener, & Battaglia, 2010).

The latter approach was used in particular to analyze the prefrontal cortex activity of behaving rats, recorded during the awake epoch and during the preceding and subsequent sleep phases by Peyrache et al. (Peyrache et al., 2009). During the awake epoch the animal faced a learning task, where it had to find the rewarded arm in a Y-shaped maze; this arm was chosen according to a rule (left or right arm, or where the light is on or off) set by the operator. As soon as the rule was consistently learned it was changed. PCA-based analysis of the recorded activity showed that the activity of the learning phase was replayed during the subsequent sleep in some experimental sessions; this replay is at the basis of memory consolidation. Here, we reanalyze the same recordings, with a more sophisticated statistical approach than PCA, based on the inference of functional connectivity between the recorded cells. Our motivation is twofold. First, the use of functional couplings allows us to characterize task-related changes in the activity of the sleep epochs in a more quantitative way than with PCA, and, in addition, to identify more sessions showing replay. Second, we expect that our more precise and extended characterization of replay could make more precise the possible connection with behavioral learning sketched in the study by Peyrache et al.

Our statistical approach relies on the inference of graphical models expressing the conditional dependencies between the spiking events of the recorded cells through functional couplings. We make use of the maximum-entropy Ising model from statistical mechanics, whose parameters are tuned to reproduce the recorded firing frequencies and pairwise cross-correlations (Schneidman, Berry, Segev, & Bialek, 2006). This inference approach has been tested on several multielectrode recordings of both in vitro (Barton & Cocco, 2013; Cocco, Leibler, & Monasson, 2009; Ferrari, Obuchi, & Mora, 2017; Schneidman et al., 2006) and in vivo (Barton and Cocco, 2013; Posani, Cocco, Jezek, & Monasson, 2017) neural activity. Working with the inferred couplings rather than considering directly the correlations in the data allows us to refine the analysis of the recording and to unveil modifications in the functional couplings between the two sleep epochs (effective positive or negative potentiation), which are consistent with the functional network derived from the learning epoch. Effective potentiation is supported by a subset of the recorded cells, which we identify by means of an automatized procedure. Our findings are supported by a large-scale study of about 100 experimental sessions. Despite the variations from session to session, presumably because of the partial and random sampling of cells, we are able to identify in about 20% of sessions a potentiated group. We then investigate in the data the change in the collective firing properties of the identified potentiated groups and find a strong increase in coactivation for such groups between the two sleep epochs. Hence, these identified potentiated groups are likely to belong to task-related cell assemblies. We then analyze how much the reactivation over time of the potentiated group is related to hippocampal inputs (ripple events), known to be important for memory consolidation. In sessions where the rule has not been learned yet, reactivation can be essentially explained as a fast response to hippocampal ripples. In some of the sessions where the rule was learned, reactivation shows a strong slow dynamical component, often unrelated to ripples, which presumably reflects the existence of a potentiated prefrontal cortex (PFC) synaptic network.

## RESULTS

We have reanalyzed recordings of the activity of tens of neurons in the prefrontal cortex of five behaving rats (Peyrache et al., 2009); see Materials and Methods for more details. Each one of the 96 recording sessions is divided into three ≃ 30-minute epochs: a Task epoch in which the rat had to learn a cross-modal rule (go left, right, where the light is on, or off, in a Y-shaped maze), which was changed as soon as the rat had learned it, and two Sleep epochs, one before (Sleep Pre) and one after (Sleep Post) the Task epoch. Through spike sorting one can identify the same neurons recorded in the different epochs (Sleep Pre, Task, Sleep Post) of a session; the number *N* of neurons reliably mapped in all three epochs varies from 3 to 56 depending on the session. No mapping could be established between different sessions.

### Inference of Functional Coupling Networks

**Figure 1.**
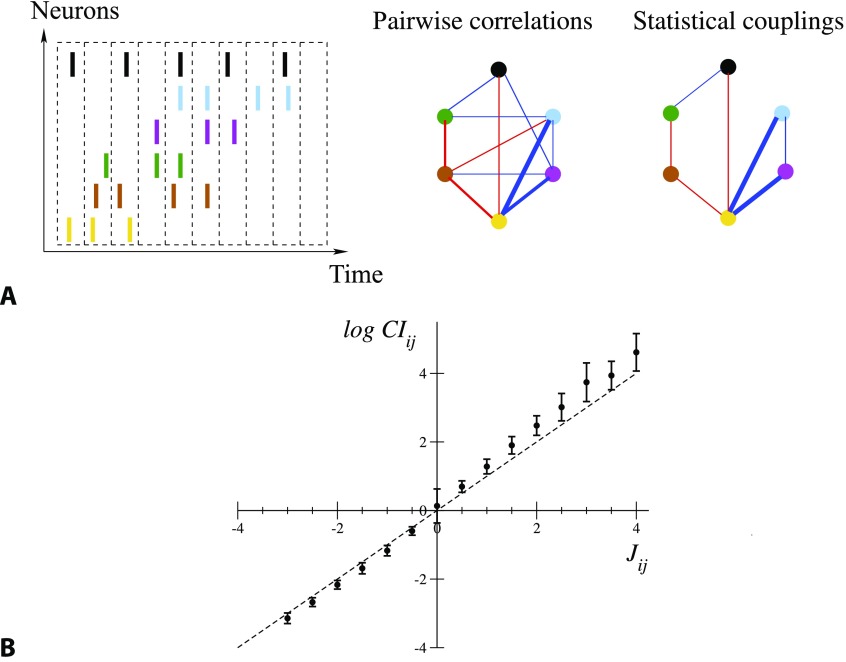
From spiking data to the Ising model. (A) Spiking times are binned into time bins of width *Δt*; each neuron *i* is assigned the variable *σ*_*i*_ = 1 or 0, depending on whether it is active in the time bin (left). Pairwise correlation indices CI_*ij*_ are computed from this binned data, and define a network of correlations (middle). The network of statistical couplings *J*_*ij*_ defining the Ising model distribution *P*, Equation 1, is generally sparser (right). Red and blue links correspond, respectively, to CI > 1, *J* > 0 and to CI < 1, *J* < 0; the widths are proportional to the absolute values. Links corresponding to CI or *J* smaller than one tenth of the maximal correlation index or coupling are not shown. (B) Average values and standard deviations of log CI_*ij*_ over intervals of couplings 0.5 *n* − 0.25 ≤ *J*_*ij*_ < 0.5 *n* + 0.25, with integer *n*, for all epochs and sessions. Note the large error bar in *J* = 0, corresponding to the very large number of pairs *i*, *j* carrying vanishing couplings; see Figure 1A.

We briefly present the approach to model the distribution of activity of the *N* recorded neurons; see Methods section. The spiking times are binned within small time bins of duration *Δt* = 10 ms, as illustrated in Figure 1A; see Supporting Information (SI), Section II and Figure S2, for a discussion of the time-bin choice (Tavoni, Ferrari, Battaglia, Cocco, & Monasson, 2017). The activity configuration (*σ*_1_, *σ*_2_, … , *σ*_*N*_) is a snapshot of the neural activity, where *σ*_*i*_ takes values one or zero depending on whether the *i*-th neuron is, respectively, active or inactive in the time bin. We define *f*_*i*_ and *f*_*ij*_ as the average values over time bins of, respectively, *σ*_*i*_ and *σ*_*i*_*σ*_*j*_: *f*_*i*_ represents the probability that neuron *i* is active in a time bin, and *f*_*ij*_ denotes the joint probability that both cells *i* and *j* are active in a time bin.

We model the probability distribution of activity configurations as

$$P(\sigma_{1},\sigma_{2},...,\sigma_{N})=\frac{1}{Z[\{h_{i},J_{ij}\}]}\;\exp\left(\underset{i<j}{\sum }J_{ij}\;\sigma_{i}\;\sigma_{j}+\underset{i}{\sum }h_{i}\;\sigma_{i}\right),$$ (1)

where *Z* ensures normalization of the distribution. *P* in Equation 1, called Ising model in statistical physics, is the least constrained (with maximum entropy), default probability distribution reproducing these low-order spiking statistics (Schneidman et al., 2006). We look for the Ising model parameters {*h*_*i*_, *J*_*ij*_} such that *f*_*i*_ and *f*_*ij*_ match, respectively, the average values of *σ*_*i*_ (for all neurons *i*) and *σ*_*i*_*σ*_*j*_ (for all pairs of neurons *i*, *j*) over *P*. To do so we use the adaptive cluster expansion (ACE) inference algorithm (Barton and Cocco, 2013; Cocco & Monasson, 2011, 2012), which also gives access to the statistical uncertainties {*δh*_*i*_, *δJ*_*ij*_} over the inferred parameters (Methods). Parameters *h*_*i*_ define effective local inputs that tune neuronal frequencies. Parameters *J*_*ij*_ define the effective pairwise couplings between the cells (Figure 1A): *J*_*ij*_ different from zero expresses the presence of a conditional dependence between neurons *i* and *j*, not mediated by other neurons in the recorded population. The conditional average activity of neuron *i* given the other neuron activities {*σ*_*j*_}, with *j*≠*i*, reads

$$\langle \sigma_{i}\rangle =\frac{P(\sigma_{1},\ldots ,\sigma_{i}=1,\ldots ,\sigma_{N})}{P(\sigma_{1},\ldots ,\sigma_{i}=0,\ldots ,\sigma_{N})+P(\sigma_{1},\ldots ,\sigma_{i}=1,\ldots ,\sigma_{N})}=\frac{e^{V_{i}}}{1+e^{V_{i}}}\;,\;\text{with}\;V_{i}\equiv \underset{j(\neq i)}{\sum }J_{ij}\;\sigma_{j}+h_{i}\;.$$ (2)

It is a logistic function of its total input, *V*_*i*_, equal to the sum of the other neuron activities *σ*_*j*_ weighted by the couplings *J*_*ij*_, and of the local input *h*_*i*_.

Though effective couplings *J*_*ij*_ are abstract quantities defined through Equations 1 and 2 they can be approximated by the logarithms of the correlation indices, CI_*ij*_ = *f*_*ij*_/(*f*_*i*_*f*_*j*_). As shown in Methods, in the simple case of *N* = 2 recorded neurons only, *J*_12_ and log CI_12_ are equal. For *N* ≥ 3, log CI_*ij*_ is only an approximation to *J*_*ij*_, and their difference quantifies the indirect contributions to pairwise correlations, mediated by other cells and not due to direct interactions; see Methods. Figure 1B shows that this approximation is good for most couplings in the recorded sessions, but deviations can be observed in particular for large and positive correlation indices; see Barton and Cocco (2013) for a discussion of the differences between log CI_*ij*_ and *J*_*ij*_ across various neural datasets. Note that the functional networks are sparse: A large fraction (about 75% over all epochs and sessions) of the couplings are regularized to zero by the inference procedure.

**Figure 2.**
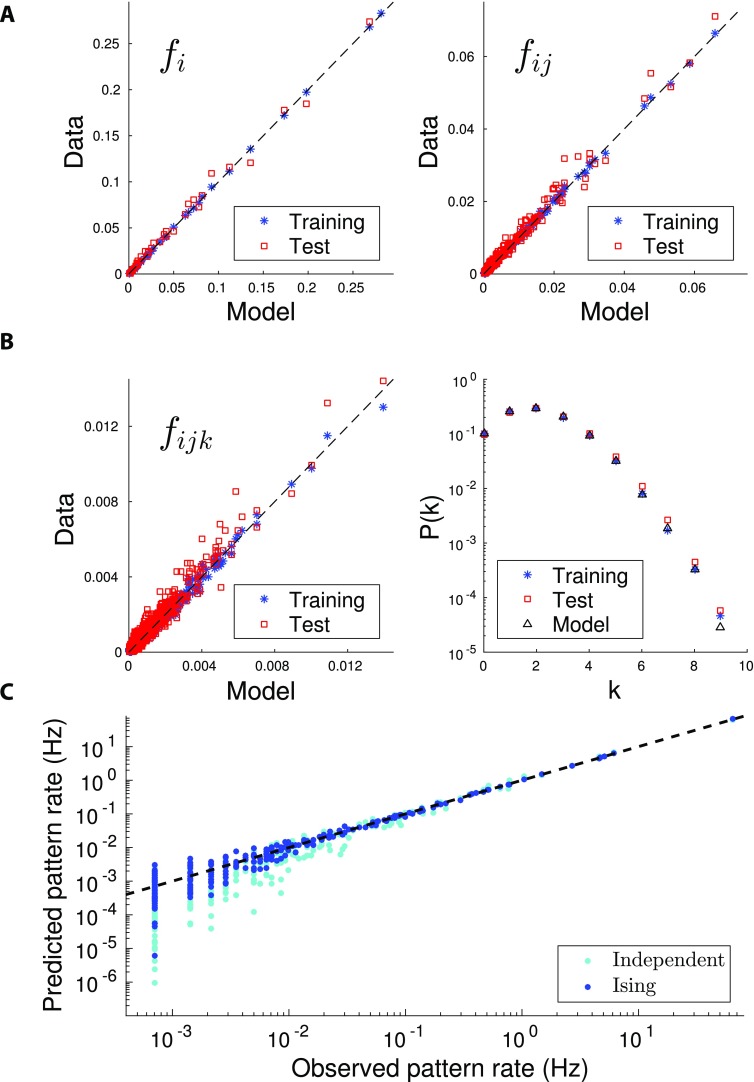
Quality and validation of the inferred model. Reproduction of the statistics of the spiking data for the Task epoch of session A. All panels compare the values of the observables computed from the spiking data with their counterparts computed from the inferred model distribution *P*, Equation 1. (A) Firing probabilities of single (*f*_*i*_, left panel) and pairs of (*f*_*ij*_, right panel) neurons. The agreement between the spiking probabilities computed from the data and from the inferred Ising distribution shows that the inference procedure is accurate. Model distribution *P* was inferred from three fourths of the recorded data and tested on the same data (blue cross) and on the remaining one fourth of the recording (cross-validation, red squares). (B) Probabilities of firing for triplets (*f*_*ijk*_, left panel) of neurons, and of *k* neurons to be simultaneously active in a time bin of duration *Δt* = 10 ms (right panel). The agreement between the data and model multiple-neuron firing probabilities (*p*(*k*)) is very good as long as *p*(*k*) times the number of time bins in the recording is > 1, that is, provided the recording time is sufficient to sample rare configurations of multiple neuron firing. Same cross-validation procedure and symbols as in Figure 2A. (C) Probabilities of the 2^10^ = 1,024 activity configurations over a subset of 10 cells in the Task epoch of session A. Blue symbols show the scatter plot for the Ising distribution *P* (inferred from all recorded data), while cyan symbols correspond to the independent-cell model (with all couplings *J*_*ij*_ = 0, and local inputs *h*_*i*_ fitted to reproduce the single-neuron probabilities *f*_*i*_). Similar plots are found for other subsets of 10 cells among the *N* = 37 recorded cells.

Once the coupling and local input parameters are inferred, we may sample the model distribution *P* through Monte Carlo simulations to check how the statistics of the data are reproduced by the model. The quality of the reproduction of the single-neuron and pairwise spiking probabilities in a time bin is shown in Figure 2A for the Task epoch of one particular session, which we call A. We can then use *P* to make predictions for higher-order moments, such as triplet firing probabilities and the probability of multiple-neuron firing in a time bin. Results are compared with the same quantities computed from the spiking data in Figure 2B. The quality of the inferred distribution *P* is then assessed through a cross-validation procedure: We divide the dataset into a train set (three fourths of the time bins) and a test set (one fourth of the bins). The good agreement between the values of observables in the train and test sets in Figures 2A and B confirms the absence of overfitting in our inference (see Figure S3 in Tavoni et al., 2017, for results on the other three out of four possible ways to define training and testing sets). We show in Figure 2C the probabilities of the 2^10^ configurations of firing of one subset of 10 cells. The Ising model predictions are in much better agreement with the data than the independent-cell model, which reproduces the single-neuron spiking probabilities *f*_*i*_ only. Taking into account pairwise correlations through the effective couplings *J*_*ij*_ is therefore crucial to better fit the neural activity distribution.

### Comparison of Functional Couplings Across Epochs Shows Learning-Related Potentiation

**Figure 3.**
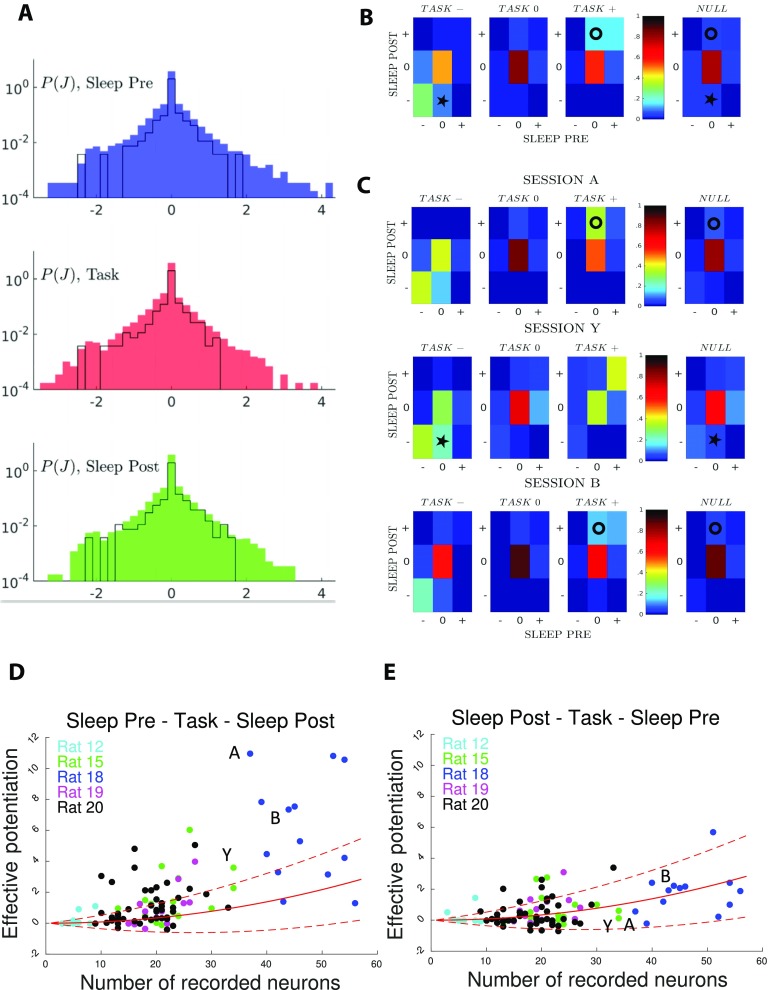
Comparison of couplings across epochs shows task-related effective potentiation. (A) Distributions of the inferred couplings *J* across the recording sessions for the three epochs (from top to down, Sleep Pre, task, and Sleep Post). The black lines show the histograms for session A only. The sharp peaks in *J* = 0 in the coupling distributions show that the inferred coupling networks are sparse. The average fractions of vanishing couplings are close to 0.75 in all three epochs. The part of the *J* distributions corresponding to the minimal value of couplings (≃−2) permitted by the regularization is due to pairs of cells that never spike together (*f*_*ij*_ = 0); see Methods. (B) Fractions of pairs of neurons (*i*, *j*) in the 27 classes [*xyz*], where *x*, *y*, and *z* = −,0,+ are the signs of the inferred couplings *J*_*ij*_ in, respectively, Sleep Pre, Task, and Sleep Post. Fractions are computed from all epochs in the 96 recorded sessions. Rightmost column: null model for the fractions corresponding to classes *x*, *z* in the Sleep epochs, irrespective of the *y* class in Task. Circle and star symbols identify classes referred to in main text. (C) Same as panel B, but for sessions A (top), Y (middle), and B (bottom). (D) Effective potentiation *Pot* (Equation 3) is shown for the 96 experimental sessions as a function of the number of recorded neurons identified in all three epochs. Colors identify the five recorded rats. Sessions A, B, Y are shown with their labels. Red lines show the effective potentiation (average: full line, ± 1 standard deviation: dashed lines) expected from the null model as a function of the number of recorded neural cells; see Methods. (E) Right: control case, where we have exchanged the Sleep Pre and Sleep Post inferred couplings.

The distributions (over all sessions) of inferred coupling parameters are similar across epochs; see Figure 3A. In addition, little variation over the magnitudes of couplings is observed from session to session. As an illustration we report in Figure 3 the histograms of coupling parameters for session A. Because of the smaller number of data, the histograms are less smooth than the average distribution over all sessions, but span the same ranges of values for *J*. Similar results hold for local inputs; see SI, Figure S4 (Tavoni et al., 2017).

Despite the overall similarities between the coupling distributions across epochs, subtle patterns can be observed when tracking the changes in the couplings corresponding to the same pairs of cells across the different epochs of the same session. We partition the set of couplings in each epoch into three classes, according to their values *J* and statistical uncertainty *δJ*: Couplings reliably inferred as positive, that is, such that *J*/*δJ* > 3, define the [+] class; couplings reliably inferred as negative (*J*/*δJ* < −3) form the [−] class; and the remaining couplings are gathered into class [0]. Each pair of neurons (*i*, *j*) belongs to one of the resulting 27 classes; for example, [− + 0] if *J*_*ij*_ is reliably negative in Sleep Pre, positive in Task, and statistically undetermined in Sleep Post.

The fractions of pairs of neural cells in the 27 classes, averaged over all sessions, are shown in Figure 3B; see SI, Figure S6 (Tavoni et al., 2017). Because of the sparsity of the inferred functional couplings, classes with vanishing couplings, such as [000], contain most of the cell pairs. We observe the presence of conserved couplings across the three epochs, corresponding to the large fractions of pairs in classes [−−−] and in [+ + +], compared with a simple null model, in which we pull together all couplings according to their classes in Sleep Pre and Post, irrespective of the class in Task (rightmost panel in Figure 3B).

An important feature emerging from Figure 3B is the presence of task-related effective (positive) potentiation in the functional couplings. This effect is visible from the relative enrichment of [0 + +] (marked with a circle symbol) with respect to the null model (two-tail binomial test, *p* ≪ 10^−5^; see Methods), while no such enrichment is found for classes [0 − +] and [00+]. In other words, we find that the fraction of pairs of neurons with close-to-zero couplings in Sleep Pre and positive couplings in both Task and Sleep Post is larger than what would be expected from the knowledge of the coupling classes in the Sleep epochs only. Task-related effective negative potentiation, corresponding to the enrichment of [0 −−] (star symbol), is also found, but with a weaker magnitude (*p* < 10^−5^).

While the results above were obtained through averaging over all sessions, there are substantial variations in the fractions of pairs in the classes from session to session. We show in Figure 3C three examples, referred to as sessions A, Y, and B. For sessions A and B effective potentiation, represented in particular by class [0 + +] (circle symbols), is clearly visible (with, respectively, *p* < 10^−5^ and *p* = 0.002). Session Y shows a strong effective negative potentiation, represented in particular by class [0 −−] (star symbol, *p* < 10^−5^).

To characterize quantitatively experience-related changes in the functional couplings in each session, we introduce the following session-wide effective potentiation, measuring the amount of potentiation in the couplings from Sleep Pre to Sleep Post, coherently with their values in Task:

Pot=∑pairsi,jnot in [0] classesin Task and Sleep PostθJijTask−JijSleepPre×JijSleepPost−JijSleepPre. (3)

Summation is restricted to pairs *i*, *j* of neurons, whose couplings are significantly different from zero in both Task and Sleep Post (same criterion |*J*_*ij*_|/*δJ*_*ij*_ > 3 as for the classes above). The presence of the *θ* function, *θ*(*u*) = 1 if the argument *u* > 0 and 0 if *u* ≤ 0, restricts contributions to pairs, whose effective couplings increase from Sleep Pre to Task. In practice positive, respectively, negative contributions to *Pot* come mostly from classes [0 + +], respectively, [0 + −].

The effective potentiations *Pot* of all 96 recorded experimental sessions are shown in Figure 3D. In comparison we show in Figure 3E the same quantity *Pot* computed after swapping, in each session, the Sleep Pre and Sleep Post couplings in Equation 3. No session is found to have a large effective potentiation after the swap, see Figure 3E. This simple control provides clear evidence for the fact that large values of *Pot* capture experience-related changes in the Sleep Post couplings. This empirical observation can be made more precise through the introduction of a null model, in which the correspondence between pairs of neurons across the epochs is removed by reshuffling the neuron indices, and the values of the couplings are randomly drawn from the distributions of Figure 3A. Red curves in Figure 3D show the average value of the effective potentiation within this null model, together with ± one standard deviation (Methods). In the control analysis of Figure 3E, where the Sleep Pre and Post data have been swapped, the effective potentiation is compatible with the expectations of the null model. Conversely, in Figure 3D, where the causal ordering Sleep Pre–Task–Sleep Post has been maintained, some sessions have large and positive *Pot* more than one standard deviation above the null model average.

**Figure 4.**
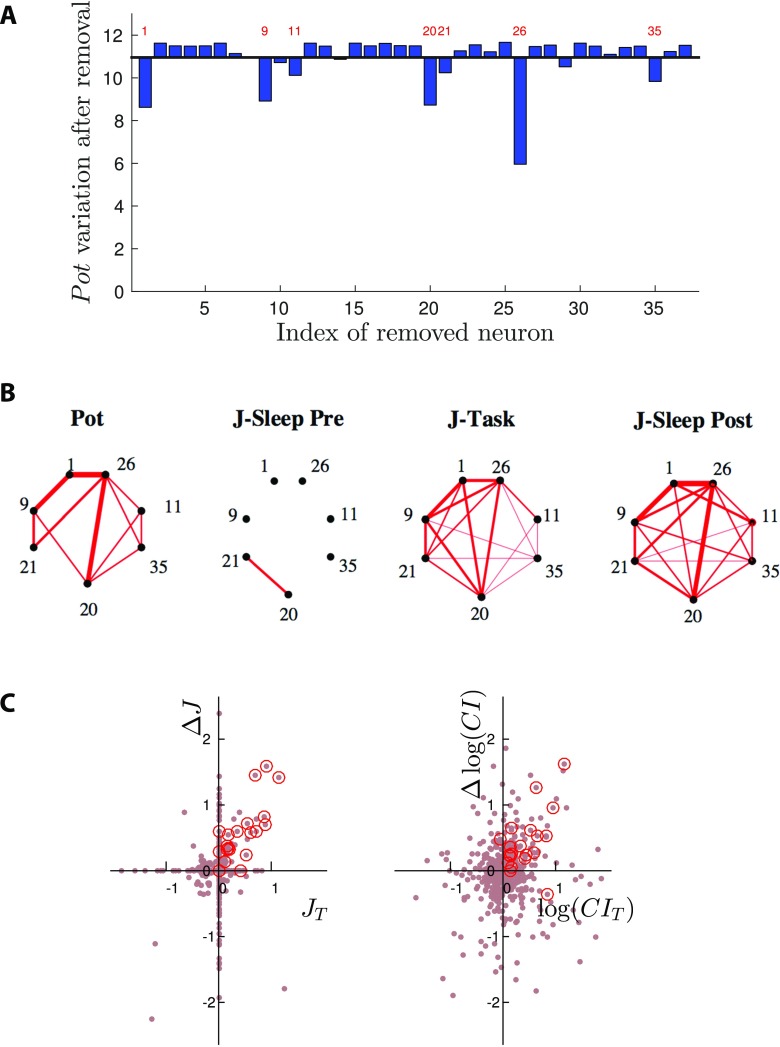
Group of neural cells supporting effectively potentiated couplings in session A. (A) Effective potentiation *Pot* after removal of one cell (index along the *x*-axis) from the spiking data of session A. For most cells the removal has no significative impact. A substantial decrease is observed for a few cells, indicated with their indices. (B) Left: main pairwise contributions *Pot*_*ij*_ to the effective potentiation (Methods). Right: Networks of couplings supported by the seven-cell group identified in panel A, in the three epochs of session A. Line thickness is proportional to *Pot*_*ij*_ (left panel) and *J*_*ij*_ (right panels). (C) Left: Scatter plot of the differences between the couplings in the Sleep epochs, $\Delta J_{ij}=J_{ij}^{Post}-J_{ij}^{Pre}$, vs. their values in Task, $J_{ij}^{Task}$. Right: same as left panel for the correlation indices CI_*ij*_; see text for definition. A group of seven neurons supports most of the couplings that are large and positive in Task and in Sleep Post, but not in Sleep Pre; red circles identify the 21 couplings between those seven neurons.

A major source of variability in the data is the limited number of randomly sampled neurons. To assess the influence of sampling on *Pot*, we focus on one particular session (A) with large effective potentiation, and remove cells, one at a time, from the recording. Results are shown in Figure 4A. In most cases removal of one cell does not significantly affect the value of *Pot*. A substantial decrease is, however, observed for a small number of cells, indicated by the labels in Figure 4A. This result clearly shows that most contributions to *Pot* come from a restricted subset of the recorded neurons. How many of those relevant cells are or are not well sampled may explain, at least in part, the variability in potentiation values observed across sessions.

### Groups of Neurons Supporting Effective Potentiation Are Replayed in the Sleep Epoch After Learning

The network of couplings supported by the group of neurons identified in Figure 4A are shown for the three epochs of session A in Figure 4B. The effective potentiation from Sleep Pre to Sleep Post and the strong similarity between the densely interconnected networks in Task and Sleep Post are clearly visible. For this session the potentiated couplings are not supported by independent, nonoverlapping pairs of neurons, but are densely interconnecting a restricted group of neurons (see Figure SI in Tavoni et al., 2017, for statistical validation). We emphasize that experience-related change in the correlational structure of Sleep Post is better seen with effective couplings than with pairwise correlations. For session A again, we show in Figure 4C (left) the variations of the couplings between the Sleep epochs, $J_{ij}^{Sleep\;Post}-J_{ij}^{Sleep\;Pre}$, versus their values in the Task epoch, $J_{ij}^{Task}$. Most contributions to *Pot*, located in the top right quadrant, are supported by the 7 cells identified in Figure 4A (red circles in Figure 4C). This shows again that the changes experienced by the couplings *J*_*ij*_ between the Sleep epochs are positively correlated to their values in Task. Conversely, the same comparison with the CI instead of the couplings *J* shows a much blurrier picture; see Figure 4C: The changes in CI between the Sleep epochs do not seem correlated with their values in Task.

While removing one cell at a time is an effective procedure to determine which neurons contribute most to *Pot*, it is computationally demanding. We have therefore developed a fast and fully automatized spectral procedure to directly identify in each session the group of neurons supporting the densest core of strongly potentiated couplings. Our procedure is based on taking the neurons with largest entries in the top eigenvector of the *Pot* matrix, whose elements are the contributions to *Pot* (Equation 3) of the pairs (*i*, *j*); see Methods for details. The top eigenvectors are shown in SI, Figure S9 (Tavoni et al., 2017), for a few sessions. We generally observe a few large entries, and many small ones. We have set a conservative value for the threshold to retain only large entries, see Methods and SI (Tavoni et al., 2017). For session A, our automatized procedure finds the five cells (the seven neurons identified in Figure 4A, but neurons 11 and 35) that support the couplings contributing most to *Pot*. This result extends to other sessions: We find a high correlation (0.76 ± 0.26 across all sessions) between the top eigenvectors of the *Pot* matrices and the “leave-one-out” potentiation vectors (shown in Figure 4A for session A), which require much more computational efforts.

We now show that the groups of neurons identifed with our automatized procedure across the 96 sessions really coactivate in the spiking data. To this aim we consider an extension of the pairwise correlation index CI_*ij*_ to groups of more than two neurons. We define the assembly coactivation ratio (CoA) of a group *G* of neurons over the time scale *τ* through

$$\text{CoA}(G,\tau )=\frac{f(G)}{\underset{i\in G}{\prod }f_{i}},$$ (4)

where *f*(*G*) is the probability that all the neurons in the group are active within the time scale *τ*, and the denominator is the product of the individual spiking probabilities. For a group of independent cells the CoA is on average equal to unity. CoA is a very stringent measure of coactivation, as it takes into account only events in which all the neurons in the potentiated group are active. A less restrictive measure of the activity of the potentiated group will be studied in the next section.

**Figure 5.**
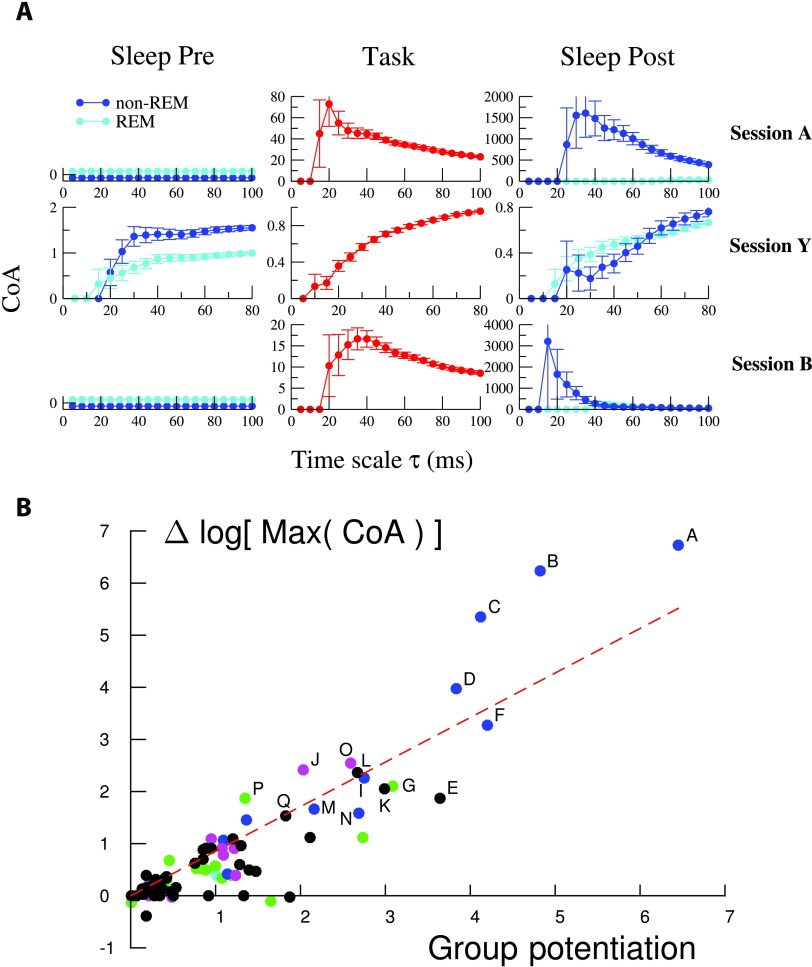
Neurons supporting effectively potentiated couplings show strong increase in coactivation. (A) Assembly coactivation ratio (CoA) for groups supporting the effectively potentiated networks of sessions A, Y, and B. Light and dark blue curves show the CoA for, respectively, the REM and non-REM periods of the Sleep epochs. CoAs are shown for time scales *τ* ranging from 5 ms to *n* × 20 ms, where *n* is the number of neurons in each group considered. Note the variations in the CoA and temporal scales along the *y*- and *x*-axis between the panels. See Methods for the computation of error bars. Note that values of CoA smaller or equal to unity can be compatible with the independent-cell hypothesis when neurons have very low firing rates; see Methods. The potentiated groups of sessions A, B, and Y include, respectively, five, five, and four neurons. (B) Logarithmic change in the peak CoA of the identified potentiated group between Sleep Pre and Sleep Post vs. potentiation *Pot* for all experimental sessions with at least two cells in the potentiated group. The sessions labeled A–Q have a large group potentiation and a large logarithmic change in their peak CoA (Δ log Max (CoA) > 1.5). The straight line shows a linear fit of the results (slope = 0.85, *R*^2^ = 0.8, *p* value = 10^−32^), proving that large *Pot* correspond to strong differences in coactivation in the spiking data between the Sleep Pre and the Sleep Post epochs.

We first show in Figure 5A the CoA of the five-cell potentiated group of session A above. This five-cell group is found to strongly coactivate in Task on a *τ* ≃ 20 − 40 ms time scale, and in Sleep Post on a similar time scale, *τ* ≃ 30 − 50 ms. The five-cell group does not coactivate more than expected by chance in Sleep Pre, which is compatible with the independent-cell hypothesis due to the low firing frequencies (Methods). This result shows that the potentiated group is replayed in Sleep Post. Interestingly, the coactivation of the potentiated group in Sleep Post is much stronger during non-REM-Sleep periods(non-REM), in which hippocampal sharp waves are known to be important for memory consolidation (Figure 5A, right). In addition, the large CoAs of the potentiated group found in Task and Sleep Post are significantly higher than CoAs for random groups of five neurons (SI, Figure S15; Tavoni et al., 2017). Those findings suggest that the five-cell group is (part of) a cell assembly that is reinforced by experience.

For each recorded session we then measure the maximal values (over the time scale *τ*) reached by the CoA of the group supporting the effectively potentiated couplings in the Sleep Pre and Sleep Post epochs. The ratio of the maximal CoA in Sleep Post over the maximal CoA in Sleep Pre is a measure of the reinforcement of the coactivation between the neurons in the group across the two sleep epochs in a session. Figure 5B shows the scatter plot of the logarithms of the ratios of those two maximal CoA versus the values of the effective potentiations *Pot* of the groups (defined as the sums of contributions to *Pot* over the pairs of neurons in the groups) across the recorded sessions. A clear monotonic trend is observed, showing that our estimate of coupling potentiation is a good estimator of the existence of neural groups in the spiking data, which reinforce their coactivation in the sleep epoch following task-learning. We retain all the sessions in which the increase in the logarithms of maximal CoA across the Sleep epochs is larger than 1.5, and label them with letters A to Q; see Figure S7 (Tavoni et al., 2017) for locating sessions A–Q in the potentiation results of Figure 3D . The sizes of the potentiated groups in sessions A–Q range from two to seven cells. It is important to notice that variants of the identified potentiated groups with, say, one more or less cells, can also have large CoAs. Varying the threshold used in the spectral procedure allows us to explore these alternative groups in each session. Examples are provided in SI, Figures S13–S14 (Tavoni et al., 2017).

While we have focused above on effective potentiation corresponding to an increase of the couplings across the Sleep epochs, effective negative potentiation, in which couplings get more negative in Sleep Post than in Sleep Pre, may be found in some sessions, such as Y (Figure 3C), despite being weak on average (Figure 3B). An analysis of the [0 −−] coupling class in session Y has permitted us to identify a cluster of three cells. We show in Figure 5A that this three-cell group is associated with a decrease of the CoA from Sleep Pre to Sleep Post in non-REM and at short time scales *τ* ∼ 20 − 40 ms. Equation 3 for *Pot* can be straightforwardly modified to define the effective negative potentiation; see SI, Section VIII and Figure S16 (Tavoni et al., 2017). In addition to Y we have identified another strongly negatively potentiated session, Z, and two sessions, C and I, showing both positive and negative potentiations across their coupling networks. As it is statistically hard to reliably estimate low CoA values, a systematic study of negative potentiation across all sessions is difficult, and would require longer recordings.

### Dynamics of Reactivation: Effects of Hippocampal Ripples and Connection With Behavioral Learning

The previous analysis allowed us to identify effective potentiated groups that are strongly coactivated in Sleep Post in sessions A–Q. A fundamental issue is whether the reactivation of those potentiated groups is mostly triggered by hippocampal inputs (sharp-wave ripples, monitored in the experiments; see Methods) or reflects the internal dynamics of the PFC network, modified upon learning. To address this question, for each of the above sessions, we define the Reactivation of the potentiated group in time bin *t*, $R(t)=\frac{1}{K}\sum_{\ell =1}^{K}\sigma_{i_{\ell }}(t)$, where (*i*_1_, *i*_2_, … , *i*_*K*_) are the indices of the *K* neurons in the group, and the average value of the reactivations over all time bins, 〈*R*〉. Reactivation *R*(*t*) is less restrictive than CoA in Equation 4 as it does not require the synchronous coactivation of all the neurons in the group.

**Figure 6.**
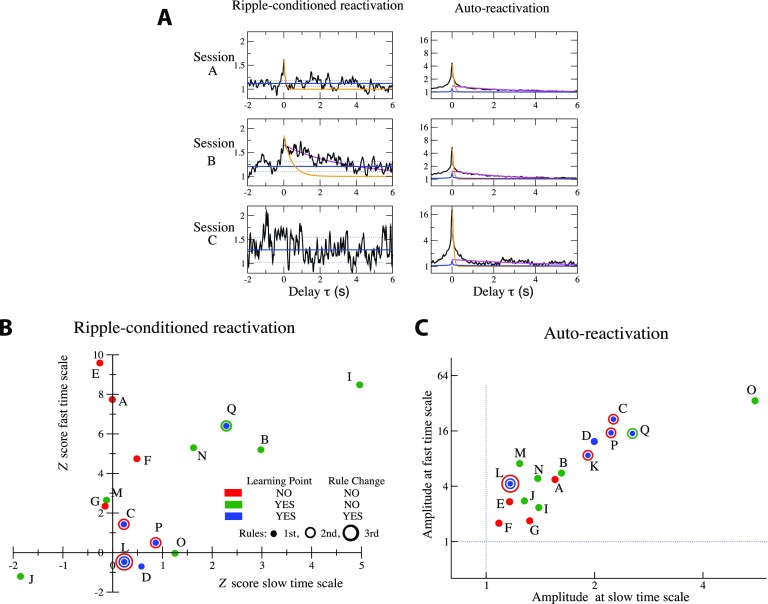
Ripple-reactivation and auto-reactivation of potentiated groups and learning behaviors. (A) Sliding average over a 50 ms time window of the ripple-conditioned reactivation (*RR*(*τ*) in Equation 5, left panels) and auto-reactivation (*AR*(*τ*) in Equation 6, right panels) of the potentiated groups for sessions A, B, and C. Orange and magenta lines show, respectively, exponential fits of the fast (over the 20 < *τ* < 100 ms range) and slow (over the 1 < *τ* < 4 s range) components to *RR* and *AR*. Fast decay times are so fitted to *τ* = 85 ms and *τ* = 400 ms for, respectively, sessions A and B, and the slow decay time for session B is *τ* = 3.5 s. Left: blue lines represent the null model for *RR* (full line: average value of *RR*, dotted lines: ± 1 standard deviation); see Methods. Right: blue lines show for comparison the *AR* curve of the groups of the same size as the potentiated groups and containing the most active neurons in each session. (B) *Z* scores *Z*(*τ*), Equation 11 in Methods, of the amplitude of the fast (*τ* = 0) and the slow (averaged over 0.5 s < *τ* < 1.5 s) components to the ripple-conditioned reactivation for sessions A to Q. Each session is represented by one, two, or three circles of increasing diameters, depending on the number of rules. For each rule the color of the corresponding circle indicates whether the learning-point and rule-changing criteria were reached. Session K is not shown because of the very small number of detected ripples. In session D, both learning-point and rule-changing criteria were met but the rule was not changed in the experiment as it should have been; see SI, Section IX (Tavoni et al., 2017). Average and standard deviation of the fast decay times over the eight sessions with largest fast responses to ripples are *τ*^*RR*−*fast*^ = 230 ± 180 ms. Average value and standard deviation of the slow decay time over the three sessions (I, B, Q) with a significant slow response to ripples are *τ*^*RR*−*slow*^ = 4 ± 3 s. (C) Amplitudes of the fast (in *τ* = 0) and the slow (average over 0.5 s < *τ* < 1.5 s) components in *AR*(*τ*) for sessions A to Q. Same color code for learning behavior as in panel **B**. Fast and slow decay time constants, fitted over all sessions are, respectively, *τ*^*AR*−*fast*^ = 56 ± 24 ms and *τ*^*AR*−*slow*^ = 3 ± 1.5 s.

We first compute the ripple-conditioned reactivation, *RR*(*τ*), defined as the average value of the reactivation following a ripple event by a delay *τ*, normalized by the average reactivation,

$$RR(\tau )=\frac{1}{N_{r}\;\langle R\rangle }\overset{N_{r}}{\underset{m=1}{\sum }}R(\tau +t_{m}),$$ (5)

where the *t*_*m*_s are the times of the *N*_*r*_ ripple events. Figure 6A shows the ripple-conditioned reactivations *RR*(*τ*) for the Sleep Post epochs of sessions A, B, C, which are representative of the variety of *RR* patterns found across all sessions. In sessions A and B, a marked reactivation peak is found at short time scales of tens of milliseconds. In addition, this “fast” peak is followed in session B by a long-lasting reactivation, decaying over a few seconds. We stress that fast versus slow reponses to ripples were not studied session by session in Peyrache et al. (2009), which reports only the average response over all sessions. No clear response of the reactivation to ripple events is found in session C on any time scale.

A complementary characterization of the reactivation dynamics, not directly related to ripples, is provided by the following normalized autocorrelation of the reactivation:

$$AR(\tau )=\frac{\langle R(t)\;R(t+\tau )\rangle }{{\langle R\rangle }^{2}},$$ (6)

where the brackets 〈⋅〉 denote the average over all time bins *t*. *AR* captures dynamical scales, irrespective of their origins (inputs from the hippocampus or internal dynamics of the PFC network). The behaviors of *AR*(*τ*) are reported for sessions A, B, C again in Figure 6A. For all three sessions we observe a large peak in the autocorrelation at *τ* = 0, expressing the tendency of neurons in the potentiated group to fire together and decaying over a few tens of milliseconds. This “fast” peak is followed by a slow component decaying over few seconds. Remarkably, in session C, for which no reactivation following ripples was detected, *AR* is stronger than for sessions A and B.

The results above were extended through a systematic analysis of sessions A–Q showing strong effective potentiation. The magnitudes of the fast (0 < *τ* < 50 ms) and slow (0.5 s <*τ* < 1.5 s) components of the ripple-conditioned reactivation are reported in Figure 6B; results are expressed in terms of *Z* scores with respect to a null model defined from the behavior of *RR*(*τ*) at negative delays *τ* < 0; see Methods. In Figure 6C we plot the amplitudes of the fast and slow components to the autocorrelation *AR*(*τ*).

We now attempt to relate the characterization of sessions in terms of *RR* and *AR* to the learning behavior of the rat during the Task epoch. The experimental protocol is described in Methods and in SI, Section IX (Tavoni et al., 2017); see also Peyrache et al. (2009). The rule was changed during the session if the rat had done 10 consecutive correct trials, or made only one error out of 12 successive trials. In most of the selected sessions A–Q, this rule-changing criterion was never reached. In some of the selected sessions, the rule-changing criterion was reached once, and a second rule was introduced; in one session (L), the criterion was met again after the second rule was set, and a third rule was introduced; see SI, Section IX (Tavoni et al., 2017).

In addition, during offline data analysis (Peyrache et al., 2009), a learning point was defined, based again on the success rate of the rat but according to a less stringent criterion: the rule was said to be learned if the rat had done three consecutive correct trials, and afterwards had a success rate larger than 80*%* over the remaining trials (up to the end of the session end or up to the change of the rule).

Whether the learning-point and the rule-changing criteria were reached defined different learning scenarios, which are illustrated by sessions A, B, C studied above (Figure 6A). In session A, a new rule was introduced at the beginning of the session, and was neither changed nor learned (learning point not reached) throughout the session. In session B, the rule was the same as in the previous session, and the learning point was reached by the end of the session but the rule was not changed. In session C, the rule was also the same as in the previous session and was changed in the middle of the session since the rat had fulfilled the rule-changing criterion; the learning point was not reached for the second rule by the end of session C. These three sessions can be informally seen as three successive levels of behavioral learning: rule not yet learned in session A (none of the two criteria is reached), intermediary learning stage (success rate has reached intermediary values between the learning and rule-changing points) in session B, and first rule definitively learned (both criteria met) for session C.

Despite the limited number (16) of selected sessions, these three learning scenarios seem to be in correspondence with general features of *RR* and *AR* presented in Figures 6B and 6C (the complete list of sessions with detailed results can be found in SI, Section IX, Tavoni et al., 2017):

▪  Nine sessions (E, I, A, Q, B, N, F, M, G) have significant fast responses to ripples (*RR*, *Z* score > 2).
  –  The four sessions E, A, F, G (out of those nine sessions) in which the learning point was not reached show no slow component to *RR*.
  –  In the remaining five sessions (I, Q, B, N, M), the learning point was reached: four out of those five sessions show a significant slow component to *RR* (*Z* score > 1.5); session M is an exception (*Z* score for slow component close to zero).
  For eight of those nine sessions the rule-changing criterion was never reached; for session Q, the criterion was reached for the first rule, but not for the second rule.
▪  Seven sessions (O, C, P, D, K, J, L) show no significant response to ripples, neither on the fast nor on the slow time scales; see Figure 6B. Five out of those seven sessions (O, C, P, D, K) show both large fast and slow components in the autocorrelation of the reactivation (*AR*); see Figure 6C (J and L show neither large *RR* nor large *AR*). In four of those sessions (C, P, D, K) the rule-changing criterion was reached. The exception is session O, which, however, presents some atypical features of long-lasting learning; see SI, Section IX (Tavoni et al., 2017).

In summary, these observations point to the following possible connection between behavioral learning and the features of the ripple-conditioned reactivation and the auto-reactivation of the neural group identified from the spiking data: (a) When neither the learning-point nor the rule-changing criteria are met, *RR* shows a fast component, and no slow component, while *AR* is weak, suggesting that the reactivation of the potentiated group is only due to the ripple inputs; (b) When the learning point is reached but the rule-changing criterion is not met, *RR* shows both fast and slow components while *AR* is weak, suggesting the presence of weak, underlying synaptic potentiations able to sustain the activity of the group after its initiation by ripples; (c) When both learning-point and rule-changing criteria are reached, there is no significant *RR*, but *AR* is strong; this is compatible with the fact that those sessions have been recorded at a stage of advanced learning, when reactivation of consolidated task-related cell assemblies might have become independent of hippocampal inputs. We emphasize that these observations are highly empirical and speculative. More statistics would definitively be needed to firmly establish and confirm those rules.

## DISCUSSION

In the present work we have focused on experience-related modifications to the functional connectivity matrix (between tens of recorded neurons) in the prefrontal cortex (PFC) of rats (Peyrache et al., 2009, 2010). Functional connectivity was defined through the introduction of a graphical (Ising) model, accounting for the statistical dependencies betweens spiking events of the neurons in the recorded population. Comparing the functional networks in the two Sleep epochs before and after learning we found, in a substantial fraction of the sessions under investigation, some changes correlated to the functional connectivity during the learning epoch itself. In most of these sessions, we found that a fraction of the couplings became effectively potentiated, and that those couplings were supported by a limited subset of the neuronal cells (the so-called potentiated group). In other words, a group of cells became much more strongly interconnected in the Sleep epoch after learning than before. We have directly verified on the spiking data that neurons in the identified potentiated groups coactivated much more in the Sleep epoch after than before the learning epoch, which is reminiscent of the notion of cell assembly introduced by Hebb (1949) as the basic unit of neural computation and memory. Study of the reactivation dynamics of the potentiated groups allowed us to separate effects due to hippocampal inputs (ripples) or to a putative PFC network, in connection with learning.

#### Patterns of Changes in Functional Couplings Between Epochs and Potentiated Groups

As a general result we have found that functional couplings define sparse interaction network in each single epoch, the class [000] concentrating most of the pairs. In addition there is an overall correlation between the amplitude of couplings across the different epochs, including Task, which can be seen from the relatively large fractions of pairs in classes [−−−] and [+ + +] compared with other nonsparse classes. Classes [0 + +] and, to a lesser extent [0 −−], contain on average significantly more pairs of neurons than, respectively, [+ + 0],[0 − +], and [−− 0],[0 + −], leading to the general conclusion that some effective couplings undergo substantial Task-related changes from Sleep Pre to Sleep Post. Notice that the classes [+ + +] and [−−−] corresponding to modulations in the amplitudes of the couplings (keeping a fixed sign) across the epochs also contribute to, respectively, effective positive and negative potentiation. These effects can be analyzed in detail, session by session.

While most of our analysis was focused on experience-related modifications to the functional connectivity, other mechanisms may take place. Tonni and Cirelli (2006) have suggested that, during specific phases of sleep (Genzel, Kroes, Dresler, & Battaglia, 2014), small synaptic interactions are erased, a phenomenon called homeostasis. The overall similarity between the distribution of inferred couplings in the two Sleep epochs, with many zero couplings, is somewhat in agreement with this hypothesis. However, it is difficult to distinguish between small couplings and couplings strictly equal to zero; homeostatic changes, if any, would likely fall in the most populated [000] class.

A potential bias in our analysis is that neither the non-REM nor the REM periods have equal durations in the Sleep Pre and Post epochs of the same session (Figure S1, Tavoni et al., 2017). We have checked the robustness of our estimates for the session-wide effective potentiation, Figure 3D, and for the group potentiation, Figure 5B, under random uniform subsampling of the recorded data in which the duration of the non-REM and REM periods were matched between the two Sleep epochs; see SI, Figure S11 (Tavoni et al., 2017).

The changes in the inferred networks of functional couplings between the Sleep epochs, correlated with the coupling network in the Task epoch, are supported by a subset of the recorded neurons. The identification of these potentiated groups of neurons was done through an automatized spectral analysis of the *Pot* matrix. We have shown that the groups of potentiated neurons strongly coactivate in the Sleep epoch posterior to learning, and are therefore part of a replayed experience-related cell assembly. It is clear, however, that the notion of potentiated group should be intended in a statistical sense. Slight variations in the composition of the group, such as adding or removing one specific neuron, are associated with large coactivation, as shown in SI, Figures S12 and S14 (Tavoni et al., 2017).

It is a remarkable and somewhat counterintuitive fact that the network of couplings inferred from pairwise coactivation on short time scales, *Δt* = 10 ms, suffices to predict coactivation patterns between *n* neurons on longer time scales, *τ* ≃ *n* × *Δt* ms. However, even in the case of coactivation events, the repeated spiking of neurons in short bursts generates a sequence of pairwise coactivation events (Figure 1A), and the coactivated groups appear as strongly interconnected. Robustness of predictions against the global temporal scale of the cell assembly and the activation ordering is an important advantage of the Ising model, because cell assembly can be played and replayed at different time scales (and in direct and reverse orderings).

#### Functional Couplings: Consequence of Common Inputs or Real Interactions?

As first discussed in the works of Gerstein and collaborators (Aertsen et al., 1989; Gerstein & Perkel, 1969), functional couplings can reflect either synaptic connections or the presence of a transient common input coactivating two or more neurons. Within the Hebbian paradigm, coactivation is a prerequisite to learning, favoring synaptic potentiation, such as through LTP (Buzsáki, 2015). In our data, common inputs could be identified in the transient sharp waves from hippocampus to the prefrontal cortex, occurring preferentially during non-REM sleep. Sharp-wave ripples have been experimentally demonstrated to be essential for memory consolidation (Buzsáki, 2015; Genzel et al., 2014; Girardeau, Benchenane, Wiener, Buzsáki, & Zugaro, 2009). Synaptic potentiation in the cortex has been suggested to take place in the immediately following stage, thanks to spindle oscillations contributing to the shutdown of the transmission from the hippocampus to the prefrontal cortex. The calcium influx taking place during spindle oscillation could facilitate synaptic potentiation between cells in the replayed assemblies (Genzel et al., 2014; Siapas & Wilson, 1998).

An important issue is whether changes in the functional couplings between the Sleep epochs reflect such common inputs, necessary for learning, or “real” plasticity in the synaptic interactions. In their earlier work Peyrache and collaborators estimated, based on principal component analysis, the average reactivation over all sessions, and showed that it occurred within a 2-second time window centered around the sharp-wave event (see Figure 5B in Peyrache et al., 2009). In our study we computed the reactivation of the more precise potentiated groups defined by the Ising model, for each one of the 16 selected sessions. In sessions with a clear response to ripple this response starts within 250 ms from the ripple event, and thus shows a finer temporal resolution. Moreover, despite the difficulty in identifying experience-related cell assemblies, the complexity of rule-changing scenarios, and the experimental limitations in the recordings, different scenarios seem to emerge, depending on the learning stage.

For the four sessions in which the rat has not learned the rule, the ripple-conditioned reactivation *RR*(*τ*) of the identified cell assembly decays after a short delay of the order of *τ* ∼ 200 ms, comparable to the typical duration of sharp-wave ripples. Hence, the strongly interconnected effective network we identified in Sleep Post (Figure 4D) seemingly mostly accounts for correlations produced by neural coactivation under common hippocampal inputs.

Furthermore, in several sessions with strong effective potentiation and in which the rule (or two rules in session Q) is (are) learned, towards the session end hippocampal ripples evoke a persistent reactivation, lasting several seconds after the ripple event. This effect may signal the existence of an established synaptic potentiation, able to reverberate the activity seconds after the input is over. In other sessions in which the rule was definitively learned and changed, no significant reactivation of the potentiated group following the ripple events was found; however, reactivation in those sessions showed a large autocorrelation, decaying over seconds.

A tentative interpretation of these findings is the following. For sessions in which the rule has not yet been learned, the large coactivation of the experience-related group, as evidenced in Figure 5A, seems to be largely supported by the inputs coming from the hippocampus during the sharp wave ripples, known to be crucial for memory consolidation. Conversely, our finding suggests that when the rule has been learned, reactivation occurs over long time scales with two possible mechanisms: slowly decaying persistence of ripple-induced activity, which is found in sessions where the rule has “just” been learned, or second-long replay periods, unrelated to ripple events, which takes mostly place in sessions where the rule has been learned and changed (more stringent criterion). The presence of long-lasting reactivation suggests the existence of a potentiated synaptic network connecting the PFC neurons. This putative network could be either evoked by ripples or subject to spontaneous excitations, depending on its maturity. A possible interpretation of the absence of ripple-induced activity for sessions in which the rule has been definitively learned is that memory has been consolidated, so ripples are not needed for the passage from short to long-term memory any longer. As stressed in the Results section, these interpretations are highly speculative, as the limited number of selected sessions and the variety of behaviors of animals during those sessions did not allow us to draw any solid statistics.

The above results are also consistent with the finding that when the rule has not been learned, the CoA of the potentiated group is often substantially larger in non-REM than in REM; see for example CoA of session A in Figure 5A. In sessions in which the rule has been learned, on the contrary, there is a still smaller but significative coactivation of the potentiated group also in REM periods, as happens in session B on the time scale of *τ* ∼ 50 ms (see also Figure S13 in Tavoni et al., 2017, for other sessions). Ripple events are indeed more frequent in non-REM sleep. Finally the presence of a CoA larger than one in Sleep Pre, which further increases in Sleep Post, is also often present when the rule is not new (see Figure S13, Tavoni et al., 2017). New experiments and more data for the different learning and rule shift protocols would be important to confirm these findings.

Note that in a related work (Tavoni, Cocco, & Monasson, 2016), we have shown how the simulation of the inferred Ising model in the presence of an external stimulation allowed us to reveal groups of coactivating cells and to characterize their statistical variations. This external input, introduced as a mathematical tool to scan rare coactivation events in the Ising model of the population activity, mimicks, in a very crude way, hippocampal inputs to the PFC and allows for the investigation of collective activation on time scales larger than the elementary time bin used for inference. The groups of neurons in the experience-related cell assemblies identified by our method and the one in Tavoni et al. (2016) coincide for the two sessions (A and D) common to our set of 16 selected sessions and to our previous work.

#### Comparison Between Inference Procedures for Ising Model

The couplings defining the graphical model considered in this paper are an extension of the correlation indices first used to quantify functional connectivity (Aertsen et al., 1989; Fujisawa et al., 2008; Schwindel et al., 2014). Informally speaking, couplings can be viewed as a sparse set of “direct” correlations among the population of recorded cells. Even for sessions with few recorded cells, the network of couplings is much sparser than its correlation counterpart (see Figure 1A). We have resorted here to the graphical Ising model, which is the maximum entropy model reproducing the one- and two-cell firing statistics. In this probabilistic framework, different methods exist to infer the couplings parameters.

A competitive inference technique is the standard Boltzmann machine learning algorithm (Hinton & Sejnowski, 1986), broadly used in the analysis of retinal (Schneidman et al., 2006) and cortical (Marre, El Boustani, Frégnac, & Destexhe, 2009) multielectrode recordings. This inference procedure is slow in its naive version but can become efficient with good initial guess of the coupling parameters (Barton and Cocco, 2013), upon replacement of the gradient descent for the minimization of the cross-entropy with an approximate version of the Newton method (Ferrari, 2016), or thanks to improvement specific to the sparse activity of neural population (Broderick, Dudik, Tkacik, Schapire, & Bialek, 2007). Two other promising methods to fit Ising models from data are the pseudolikelihood approach (Aurell & Ekeberg, 2012) and minimal probability flow (Sohl-Dickstein, Battaglino, & DeWeese, 2011). Both approaches use all the data, and not only the first and second moments of the neural activity, to avoid computing the normalization constant *Z* in the distribution *P*; see Equation 1. In particular, the minimal probability flow method has been recently applied to multilayer restricted Boltzmann machine to model explicitly the different columns in cortical data (Köster, Sohl-Dickstein, Gray, & Olshausen, 2014).

Here we have used the adaptive cluster expansion (ACE; see Methods and Barton & Cocco, 2013; Cocco & Monasson, 2011), which has been shown to accurately reproduce interaction parameters for synthetic data. The Ising distribution inferred with ACE also reproduces the statistics of retinal or hippocampal recordings of the activity of tens to hundreds of recorded neurons, including high-order moments (Barton & Cocco, 2013; Posani et al., 2017). More recently this technique was generalized to the case of nonbinary but multiple-categorial variable, or Potts model, to model coevolution in protein sequences (Barton, De Leonardis, Coucke, & Cocco, 2014). Our inference approach is very fast on these neural data, taking some seconds on a personal computer to infer the input and functional connectivity parameters. It would be possible to use it to identify cell assemblies online. Combined with optogenetics techniques (Reutsky-Gefen et al., 2013) this would open exciting perspectives on the manipulation of cortical cell assemblies in a controlled way.

The Ising model captures the statistics of snapshots of the activities, and, as such, defines symmetric functional couplings *J*_*ij*_ = *J*_*ji*_. It can therefore not be used to study the ordering in the dynamical activation of the neurons. Other functional-connectivity-based inference approaches, such as the generalized linear (Pillow et al., 2008; Truccolo, Eden, Fellows, Donoghue, & Brown, 2005), kinetic Ising (Roudi & Hertz, 2011), and integrate and fire (Koyama & Paninski, 2010; Monasson & Cocco, 2011) models are designed to infer nonsymmetric connectivity matrices from the temporal sequence of spiking events in the neuronal population. In Tavoni et al. (2016), we have inferred the couplings on the same cortical dataset with the generalized linear model and found that they are essentially symmetric, and strongly correlated with their Ising counterparts. One possible explanation is that cell assemblies in the prefrontal cortex may also code for instrinsically nontemporal aspects of the task to be learned, in agreement with the findings of Peyrache et al. (2010).

### Comparison With Existing Procedures to Identify Cell Assemblies

Many of the currently available methods to detect and characterize the replay of neural groups or cell assemblies rely on the knowledge of how the neural activity correlates with sensory or internal inputs. For example, place cells in the hippocampus are known to encode location in space, and replay of place-cell assemblies representing behaviorally meaningful trajectories can be determined with template-matching techniques. More precisely, the ordered activation sequences of place cells observed during salient moments, such as sharp-wave ripple events, during sleep or wakefulness, is matched with the sequences of place cells determined by the templates observed during locomotion (Brown, Frank, Tang, Quirk, & Wilson, 1998; Carr et al., 2011; Diba & Buzsáki, 2007; Foster & Wilson, 2006; Lee & Wilson, 2002; Pfeiffer & Foster, 2013). Similarly, in sensory systems cell assemblies can be detected and characterized by studying the neuronal population response to specific stimuli, easily reproducible in experimental settings. An example is provided by the analysis of neural activity patterns following specific sounds in the auditory cortex (Bathellier, Ushakova, & Rumpel, 2012). However, those approaches are not easily applicable to all the regions of the brain. In the prefrontal cortex, for instance, neurons may not be activated in a well-defined temporal order, predictable from the knowledge of external stimuli. Cell assemblies might respond to internal cognitive states, or to a combination of extrinsic covariates and internal states, which are very difficult to determine and control experimentally.

In this context, principal component analysis (PCA) has been used as a way to build approximate templates from the correlational structure of data (top principal components), and to detect reactivation, or replay, of those templates. Though PCA was applied successfully to detect replay (Benchenane et al., 2010; Peyrache et al., 2009, 2010), it lacks any probabilistic framework and the interpretation of the large entries of the top components is difficult, even with the use of clustering procedures, such as the assembly vector estimation Lopes-dos-Santos et al., 2013. Our analysis significantly extends the PCA of Peyrache et al. (2009, 2010), as it identifies the neurons participating to replay-related assemblies in a detailed way. Let us stress that the whole approach for computing functional connectivity and identifying cell assemblies is fully automatized, and requires spiking data only. While our approach identifies a single cell assembly that contributes the most to functional coupling potentiation, it could be easily extended to the case of more assemblies; see Methods and Fortunato (2010). Such an extension could be useful for analyzing bigger recordings in the future.

A community detection technique for cell assembly identification, exploiting the Markov stability method, was recently introduced in (Billeh et al., 2014). The method consists of finding a stable partition on a correlation graph and was tested on hippocampal and retinal data. This graph, unlike the coupling networks we infer here, is defined heuristically and does not disentangle direct (giving rise to coupling) from indirect (mediated through other neurons) correlations.

## MATERIALS AND METHODS

### Description of Experiments

Experimental methods were described in detail in Benchenane et al. (2010) and Peyrache et al. (2009) and are summarized in the following. Five Long-Evans male rats weighing 250–300 g at arrival were implanted with tetrodes in the prelimbic (PL) subdivision of the medial prefrontal cortex, and in the intermediate-ventral hippocampus. PL tetrodes were used for recording of single units: signals were band-pass filtered between 600 and 6,000 Hz, and spikes were detected whenever the filtered signal exceeded a manually set threshold. The resulting waveform (1.3 ms long) was fed into an automated spike sorting algorithm (KlustaKwik; Kadir, Goodman, & Harris, 2014). Hippocampal tetrodes were only used for local field potentials, for the detection of theta rhythms and sharp waves. Non-REM was automatically detected, based on power in the cortical delta band (1–4 Hz), hippocampal theta (5–10 Hz), cortical spindles (10–20 Hz), and speed of head motion, by means of a clustering algorithm. A quality check on sleep epochs ensures the absence of systematic biases in Sleep Pre with respect to Sleep Post; see Figure S1 (Tavoni et al., 2017).

The rats performed an attentional set shift task on a Y-maze, which is known to require the function of the medial prefrontal cortex (mPFC) in rats (Birrell & Brown, 2000). Each recording session consisted of a 20- to 30-minute sleep or rest epoch (Sleep Pre epoch) in which the rat remained undisturbed in a padded flowerpot placed on the central platform of the maze, a Task epoch, in which the rat performed the behavioral task described below for 20–40 min, and by a second sleep or rest epoch (Sleep Post epoch; same situation as in Sleep Pre) of 20–30 min. The whole recordings in the Task epoch and in the Sleep phases (both during REM and non-REM periods) were used for our inference.

Rats started each trial in the same arm (the departure arm). One of the two other (choice) arms was illuminated at random (pseudorandom schedule: runs of more than four consecutive trials with the same illuminated arm were avoided, as were repeated bouts of imposed alternation between the two arms). After that, the central platform was lowered, allowing the rat to access the choice arms. Only one of the choice arms was rewarded, according to one of four contingency rules. Two contingency rules were spatially guided (always go to the right arm, or to the left arm); the other two were cue guided (go to the illuminated arm, or to the dark arm). The rule that was employed at any given moment in time was not signaled to the rat in any way, so that the animal had to learn the rule by trial and error. Once the rat reached a criterion of 10 consecutive correct trials, or only one error out of 12 trials, the rule was changed with no further cue warning to the rat. Rule changes were extradimensional, that is, from a spatially guided rule to a cue-guided rule, and vice versa. All five rats learned in a consolidated way the right and light rules (at least 10 consecutive correct trials), whereas only two learned in a consolidated way the left task and one to go where the light is off.

### Inference of Ising Model Parameters

#### Inference procedure

We have inferred the Ising model parameters with the adaptive cluster expansion (ACE) algorithm (Barton & Cocco, 2013; Barton et al., 2016; Cocco & Monasson, 2011), available from https://github.com/johnbarton/ACE. ACE computes an approximation for the (cross-)entropy of the Ising model reproducing the following data:

$$S_{Ising}=\underset{h,J}{\min}\left[\right.-\underset{i}{\sum }h_{i}f_{i}-\underset{i<j}{\sum }J_{ij}f_{ij}+\log Z(\{h_{i},J_{ij}\})],$$ (7)

where *Z*[{*h*_*i*_, *J*_*ij*_}] is a function of the Ising parameters normalizing the distribution *P* in Equation 1. The ACE procedure recursively builds clusters *Γ* = (*i*_1_, *i*_2_, … , *i*_*K*_) of neurons with increasing sizes *K*, and selects those whose contributions *ΔS*_*Ising*_(*Γ*) to the cross-entropy exceed a threshold *Θ* (in absolute value) (Barton & Cocco, 2013; Cocco & Monasson, 2012). The total cross-entropy is approximated by the sum of the cluster entropies over the set of selected clusters, $S_{Ising}≃\underset{\text{selected}\;\Gamma }{\sum }\Delta S_{Ising}(\Gamma )$. The optimal value of the threshold, *Θ*, is chosen so that the Ising model (with parameters realizing the minimum in Equation 7) reproduces the experimental low-order statistics within the expected sampling accuracy. Choosing smaller values for *Θ* would require more computational efforts and would overfit the data.

#### Regularization and statistical error bars

To regularize the minimization problem in Equation 7 we (a) remove from the datasets neurons spiking less than 10 times in each epoch, that is, such that *f*_*i*_ < 10/*B*, where *B* is the number of time bins, that is, the duration of the recording divided by the time-bin duration *Δt*; (b) add to the right-hand side of Equation 7 a term $\gamma \underset{k<l}{\sum }J_{kl}^{2}$ penalizing large couplings. In practice we choose *γ* = 0.2/*B*. This choice ensures that the coupling between pairs of neurons that are never active together in any time bin and that would, in principle, be assigned an infinitely large negative value become comparable to the couplings between pairs of neurons with a few common spiking event across the recording.

To quantify the statistical errors on the inferred parameters, we evaluate the matrix of the second derivatives of the entropy *S*_*Ising*_ with respect to the Ising parameters {*h*_*i*_, *J*_*kl*_}, also called Fisher information matrix. The squared statistical error bars (*δh*_*i*_)^2^ and (*δJ*_*kl*_)^2^ on, respectively, the inferred local inputs *h*_*i*_ and the couplings *J*_*kl*_ are given by the diagonal elements of the inverse matrix of the Fisher information matrix, divided by the number of time bins, *B* (Cocco & Monasson, 2012). Remark that, with the addition of the regularization term, the Fisher information matrix is definite positive, and, therefore, can be inverted.

#### Relationship between couplings and correlation indices

In the case of *N* = 2 recorded neurons, only the normalization coefficient *Z* in Equations 1 and 7 reads $Z[h_{1},h_{2},J_{12}]=1+e^{h_{1}}+e^{h_{2}}+e^{J_{12}+h_{1}+h_{2}}$; the coupling between the two cells is easily obtained upon minimization over *h*_1_, *h*_2_, *J*_12_ in Equation 7, and reads

$$J_{12}=\log\;\left[\frac{f_{12}\;(1-f_{1}-f_{2}+f_{12})}{\left(f_{1}-f_{12})\;(f_{2}-f_{12}\right)}\right]≃\log\;\left[\frac{f_{12}}{f_{1}\;f_{2}}\right]\;,$$ (8)

for time bins sufficiently short, that is, such that *f*_12_ ≪ *f*_1_, *f*_2_ ≪ 1. Hence the effective coupling coincides with the logarithm of the correlation index CI_12_ (Barton & Cocco, 2013). For *N* ≥ 3 recorded neurons no simple relationship exists between couplings and correlation indices because of network effects.

### Statistical Tests on Task-Related Conditioning of Coupling Classes

In order to check that conditioning over the class of couplings in the Task epoch does impact the distribution of classes of couplings in the Sleep epochs and to give statistical support to the notions of effective potentiation and negative potentiation, we applied a two-tailed binomial test. We compute the binomial parameter from the task-unconditioned statistics (null model; see rightmost panels in Figures 3B and 3C). The *p* value is then computed as the probability under this binomial distribution of finding a number (of couplings in the class of interest) as rare, or rarer than the one found in the data. We find *p* ∼ 4 × 10^−42^ for potentiation (class [0 + +]) and *p* ∼ 2 × 10^−11^ for negative potentiation (class [0 −−]) across all sessions (Figure 3B), *p* ∼ 6 × 10^−9^ for potentiation in session A, *p* ≃ 4 × 10^−6^ for negative potentiation in session Y, and *p* ∼ 0.0021 for potentiation in session B (Figure 3C).

### Null Model for the Effective Potentiation

We define the null model for *Pot* shown in Figure 3C (red curves). We first introduce the three-coupling potentiation *p*_*ij*, *kl*, *mn*_ through

$$p_{ij,kl,mn}=J_{mn}^{\text{Sleep Post}}-J_{kl}^{\text{Sleep Pre}}\;\text{if}\;J_{ij}^{Task}>J_{kl}^{\text{Sleep Pre}},\;\frac{\left|J_{mn}^{\text{Sleep Post}}\right|}{\delta J_{mn}^{\text{Sleep Post}}}>3\;\text{and}\frac{\left|J_{ij}^{Task}\right|}{\delta J_{ij}^{Task}}>3\;,$$ (9)

and *p*_*ij*, *kl*, *mn*_ = 0 otherwise. Note that the effective potentiation in Equation 3 is obtained by summing all three-coupling potentiations *p*_*ij*, *ij*, *ij*_ with the same pairs of neurons in the three epochs of the sessions. In our null model, the coincidence between the three pairs of neurons is removed by picking up the three couplings in Equation 9 above uniformly at random among the set of couplings obtained from all sessions of all rats. The mean and the standard deviation of the reshuffled (mismatched) three-coupling potentiations *p* among all the sessions are 〈*p*〉≃ 0.0018 and *Δp* ≃ 0.066. Note that, as the distributions of couplings are similar from one session to another (Figure 2A), the distribution of potentiations obtained by reshuffling the couplings within each session only, without pooling all sessions together, gives similar results.

For a session with *N* recorded neurons, the null model distribution for *Pot* is obtained by summing $\frac{1}{2}N(N-1)$ randomly drawn reshuffled potentiations *p*. The null model average and its standard deviations are thus given by the following:

$$\langle Pot\rangle =\frac{N(N-1)}{2}\;\langle p\rangle \;,\;\Delta \;Pot=\sqrt{\frac{N(N-1)}{2}}\;\Delta p\;,$$ (10)

which define the red lines of Figure 3C (left). The null models for the controls in Figure 3C (right) are obtained with the same procedure after swapping the Sleep Pre and Sleep Post epochs in all sessions.

### Identification of the Group of Neurons Supporting the Most Effectively Potentiated Couplings

In order to identify the subgroup of neurons that supports the couplings with strongest effective potentiation in a session, we consider the contribution to *Pot* (Equation 3) coming from the pair (*i*, *j*) of neurons, *Pot*_*ij*_ ≡ *p*_*ij*, *ij*, *ij*_, cf. Equation 9. This contribution can be seen as an entry of an *N* × *N*–dimensional matrix. This matrix is sparse, square symmetric, and has large entries for neurons (*i*, *j*) supporting strongly potentiated couplings. The top eigenvector, **v** = {*v*_*i*_}, of the matrix is localized over few neurons *i*, which strongly contribute to *Pot* (SI, Figure S9, Tavoni et al., 2017). We define the potentiated group as the set of neurons *i* with components *v*_*i*_ larger than a threshold value *c* (ranging between zero and one for normalized *v*). In all the experimental sessions considered here, this simple spectral analysis gives at most one large and connected neural group. Spectral graph theory offers efficient methods for dealing with more complex data structures on larger datasets, including more than one largely interconnected group (Fortunato, 2010).

The correlation between the changes in log CoA from Sleep Pre to Post, Δ logCoA, and the potentiation of the group across all sessions (shown in Figure 5B for *c* = 0.22) varies with *c*. The best correlations are found in practice in the range 0.15 < *c* < 0.35, with *p* values ranging between 10^−30^ and 10^−35^ (SI, Figure S10, Tavoni et al., 2017). We have arbitrarily set *c* = 0.22 in between these two limits. This value is also adequate if one imposes in addition that potentiated groups should include at least three cells (SI, Figure S10, Tavoni et al., 2017).

### Statistical Significance of the Coactivation Ratio (CoA)

To assess the statistical validity of the CoA defined in Equation 4 for a group *G* of neurons, we compute the error bar on CoA, shown in Figure 5A. Assuming a Poisson distribution for the coactivation events, the standard deviation of the CoA is estimated to be CoA(*τ*)$/\sqrt{N_{G}(\tau )}$, where *N*_*G*_(*τ*) is the number of coactivation events for the cells in *G* over the time scale *τ*.

Note that simultaneous-firing events (contributing to *f*(*G*)) are unlikely to be found, and the CoA is likely to be zero, if the duration of the recording is small, for example, compared with $T_{min}=\tau /\prod_{i\in G}f_{i}(\tau )$. This happens for the five-cell potentiated group of session A for time scales *τ* ≤ 40 ms in the Task epoch, and for all the values of *τ* considered in Sleep Pre and Post in Figure 5A.

### Analysis of Ripple-Conditioned Reactivation

We define a null model for the average response to ripples after a delay *τ*, *RR*(*τ*) in Equation 5, as follows. For each session, we compute the average value, *RR*_0_, and the standard deviation, *δRR*_0_, of *RR*(*τ*) over the − 3 s < *τ* < −0.5 s range. The range of delays is sufficiently negative to exclude any inaccuracy in the determination of the ripple times *t*_*m*_. The values of *RR*_0_ and *RR*_0_ ± *δRR*_0_ are shown in Figure 6A (left panels) for sessions A, B, and C. The *Z* score of ripple-conditioned reactivation for positive delay *τ* is defined through

$$Z(\tau )=\frac{RR(\tau )-RR_{0}}{\delta RR_{0}}.$$ (11)

The value of the *Z* score in *τ* = 0 and its average over the 0.5 s < *τ* < 1.5 s interval are used to estimate the amplitudes of the, respectively, fast and slow components in *RR*; see Figure 6B.

## ACKNOWLEDGMENTS

We thank Georges Debregeas for useful comments about the manuscript. This work was funded by the [EU-]FP7 FET OPEN project Enlightenment 284801.

## AUTHOR CONTRIBUTIONS

Gaia Tavoni: Formal analysis; Investigation; Methodology; Validation; Writing original draft, first, equal; Writing – review & editing. Ulisse Ferrari: Formal analysis; Methodology; Validation; Writing – original draft; Writing review & editing. Francesco Battaglia: Conceptualization; Investigation; Project administration; Supervision; Writing – original draft, first, equal; Writing review & editing. Simona Cocco: Conceptualization; Investigation; Methodology; Project administration; Supervision; Validation; Writing – original draft, last, equal; Writing – review & editing. Rémi Monasson: Conceptualization; Investigation; Methodology; Project administration; Supervision; Validation; Writing – original draft, last, equal; Writing – review & editing.

## TECHNICAL TERMS

Functional couplings: Effective interactions between recorded units explaining the observed pattern of correlations in the recorded population neural activity.
Replay: Reactivation of task-associated cell assemblies during sleep following learning.
Cell assembly: A group of coactivating cells, which have been postulated by D. Hebb as the basis for neural computation and memory.
Principal component analysis (PCA): Statistical procedure that explains a large fraction of the variability in the data from the top eigenvalues/ eigenvectors of the Pearson correlation matrix.
Graphical models: Probabilistic models of interacting variables, in which the distribution of each variable generally depends on a restricted number of other variables.
Ising model: Mathematical model to describe systems of binary interacting components in statistical physics.
Effective potentiation: A quantity that sums up, over all the pairs of neurons, the increases of the coupling, between the sleep after the task and the sleep before the task, only if there is a correlated coupling increase between the task and the sleep before the task.
Potentiated group: A group of cells that sustain most of the potentiated couplings. This group is identified from the largest components of the top eigenvector of the potentiation matrix.
Cross-modal rule shift: An introduction of new rules that can be based on spatial (go to the left or to the right) or visual (go where the light is on or off) cues.
Adaptive cluster expansion: Technique of inference of the Ising model parameters based on an iterative construction of clusters of strongly interacting variables.
Correlation index (CI): Ratio of the joint probability that a pair of neurons are active in a time bin, and of the product of their individual spiking probabilities. For independent neurons, CI = 1.
Coactivation ratio (CoA) of a group of neurons: Ratio of the probability that all the neurons in a group are active within a time scale, and of the product of the individual spiking probabilities. CoA is a multineuron generalization of CI.
Non-REM sleep: Stages 13 of sleep characterized by absence of dreams and absence of rapid eye movements.
Sharp-wave ripple complexes: Large activity bursts in the hippocampus composed of large amplitude local field potential deflections (sharp waves) associated with high-frequency field oscillations, called ripples, generated particularly during immobility and non-REM sleep.
Ripple-conditioned reactivation (RR): Average reactivation of the potentiated group following a ripple event.
Autocorrelation of the reactivation (AR): Autocorrelation of the reactivation of the potentiated group.
REM sleep: Phase of sleep characterized by rapid eye movements and the propensity of the sleeper to dream.
